# Epstein-Barr Virus LMP1 Modulates the CD63 Interactome

**DOI:** 10.3390/v13040675

**Published:** 2021-04-15

**Authors:** Mujeeb Cheerathodi, Dingani Nkosi, Allaura S. Cone, Sara B. York, David G. Meckes

**Affiliations:** Department of Biomedical Sciences, Florida State University College of Medicine, Tallahassee, FL 32306, USA; mujeeb.cheerathodi@tmh.org (M.C.); dingani.nkosi@med.fsu.edu (D.N.); alllaura.cone@med.fsu.edu (A.S.C.); sara.york@med.fsu.edu (S.B.Y.)

**Keywords:** latent membrane protein 1, Epstein-Barr virus, Herpesvirus, proteomics, mass spectrometry, interactions, signaling, extracellular vesicles, exosomes, CD63, tetraspanin, mTOR, autophagy

## Abstract

Tetraspanin CD63 is a cluster of cell surface proteins with four transmembrane domains; it is associated with tetraspanin-enriched microdomains and typically localizes to late endosomes and lysosomes. CD63 plays an important role in the cellular trafficking of different proteins, EV cargo sorting, and vesicle formation. We have previously shown that CD63 is important in LMP1 trafficking to EVs, and this also affects LMP1-mediated intracellular signaling including MAPK/ERK, NF-κB, and mTOR activation. Using the BioID method combined with mass spectrometry, we sought to define the broad CD63 interactome and how LMP1 modulates this network of interacting proteins. We identified a total of 1600 total proteins as a network of proximal interacting proteins to CD63. Biological process enrichment analysis revealed significant involvement in signal transduction, cell communication, protein metabolism, and transportation. The CD63-only interactome was enriched in Rab GTPases, SNARE proteins, and sorting nexins, while adding LMP1 into the interactome increased the presence of signaling and ribosomal proteins. Our results showed that LMP1 alters the CD63 interactome, shifting the network of protein enrichment from protein localization and vesicle-mediated transportation to metabolic processes and translation. We also show that LMP1 interacts with mTOR, Nedd4 L, and PP2A, indicating the formation of a multiprotein complex with CD63, thereby potentially regulating LMP1-dependent mTOR signaling. Collectively, the comprehensive analysis of CD63 proximal interacting proteins provides insights into the network of partners required for endocytic trafficking and extracellular vesicle cargo sorting, formation, and secretion.

## 1. Introduction

Latent membrane protein 1 (LMP1) is the major oncogene of the Epstein-Barr virus (EBV) which has been demonstrated to induce cell transformation and immortalization and promote migration and invasion [1,2,3]. EBV-associated cancers include nasopharyngeal carcinoma (NPC), Burkitt lymphoma, Hodgkin’s disease, post-transplant lymphoma, and a subset of gastric carcinomas [4,5]. LMP1 is expressed in most EBV-associated cancers and acts as a constitutively active mimic of CD40 signaling and activates multiple signaling pathways which are involved in inducing genes responsible for apoptosis, cell cycle progression, cell proliferation, and migration [6,7,8]. LMP1 has also been shown to be incorporated into extracellular vesicles (EV) at high levels [9,10,11]. LMP1 containing EVs can exert oncogenic signaling functions on neighboring or distant recipient cells [9,11]. Transfer of the LMP1-containing EVs can activate MAPK/ERK and PI3K/Akt signaling functions within the recipient cells to enhance proliferation, migration, and invasion [9,12,13,14,15]. Collectively, these data suggest the significance of LMP1 in remodeling of the microenvironment through the transfer of virally modified EVs for cell to cell crosstalk.

Extracellular vesicles (EVs) are membrane-bound vesicles released from cells that are important orchestrators of intracellular communication events in healthy and pathological environments through the delivery of biologically functional molecular components. The ability to sort and transfer various cargoes including proteins, mRNAs, microRNAs (miRNAs), and lipids to neighboring or distant cells between cells renders EVs important therapeutic and biomarker targets [16,17]. Though the role and significance of EVs has received much attention, molecular mechanisms guiding biogenesis and sorting of different cargo is still not well understood. Multiple studies have recently shown the important roles the tetraspanin CD63 play in EV biogenesis and sorting of different cargo [18,19,20,21,22]. CD63 is a member of the tetraspanin superfamily, a cluster of cell surface associated membrane proteins with four transmembrane domains [23,24]. Tetraspanins have been shown to be involved in many cellular functions including cell motility, adhesion, differentiation, activation, immune response, and tumor cell migration and invasion. Post translational modification of CD63 results in its ability to organize and form tetraspanin-enriched microdomains (TEMs) on membranes [25,26]. The formation of TEMs allows interaction with components such as integrin molecules, immunoglobulins, proteoglycans, cadherins, and signaling molecules on the cell surface [23,25,27]. CD63 is highly enriched in late endosomal and lysosomal compartments after being endocytosed from the cell surface through the clathrin-coated vesicles [19,28,29]. The fusion of the late endosome or multivesicular bodies to the cell surface leads to the release of the intraluminal vesicles as EVs. 

In our previous studies, we found that the interaction of CD63 with LMP1 is important for LMP1 trafficking and secretion into EVs [19,21]. Knockout of CD63 using CRISPR Cas9 reduced LMP1 packaging into EVs and increased intracellular activation of mitogen-activated protein kinase (MAPK)/ERK and noncanonical NF-κB pathways. Additionally, CD63 is associated with regulation of balance between endosomal and autophagy cellular processes through LMP1-mediated mTOR signaling [30]. Furthermore, we have also demonstrated that LMP1 is in close proximity to and interacts with CD63 through affinity purification [31]. Taken together, these data show the significance of CD63 interaction with LMP1 in regulating the different cellular functions. 

Using the proximity-dependent biotin identification (BioID) method combined with mass spectrometry, we identified LMP1 interacting proteins that are involved in major cellular processes including signal transduction, EV formation, and protein trafficking [31]. Some of the LMP1 direct or proximal interacting proteins identified involved in protein trafficking and EV biogenesis are CD63, syntenin−1, ALIX, TSG101, HRS, CHMPs, and sorting nexins. LMP1 more likely modifies these interacting proteins and pathways to regulate intracellular signal transductions and other important cellular functions. Even though our lab and others have demonstrated the importance of CD63 in LMP1-mediated enhancement of vesicle secretion, EV trafficking, and signaling, very little is known about how CD63 mediates these events. Using the BioID method, we investigated and evaluated the broad CD63 interactome and how LMP1 modulates it. The BioID method has been successfully used in identifying potential protein–protein interactions, where the protein of interest is fused to bacterial biotin ligase BirA [31,32]. Upon expression in cells, the BirA-tagged protein biotinylates the proteins in close contact and immediate vicinity and therefore can be isolated using avidin-based pull-down methods and subjected to mass spectrometry for identification.

In this study, we identified roughly 1600 total proteins as potential direct or indirect proximal interacting proteins to CD63. The biotinylated proteins present in the dataset were enriched in signal transduction, cell communication, protein metabolism, and transport biological process. The introduction of LMP1 into the interactome identified proteins which were highly involved in metabolic processes and translation biological processes, while the CD63-only interacting proteins were enriched in SNARE proteins and Rab GTPases, which are involved in protein localization and endosomal/vesicle-mediated transportation biological process. These data suggest LMP1 utilizes CD63 to traffic signaling molecules through the endosomal pathway for secretion in EVs, and this might be an alternative mechanism of limiting LMP1 intracellular signaling. Taken together, our findings shed more light into the network of proteins CD63 requires for trafficking cargo through the endosomal system and further show how LMP1 modify the wider CD63 interactome.

## 2. Materials and Methods

### 2.1. Cells and Transfection

HEK293 (human embryonic kidney 293) wild-type cells and HEK293-expressing CD63 CRISPR were grown in DMEM (Delbouco’s modified eagle medium, Lonza; 12-604Q) with the following supplements: 10% FBS (fetal bovine serum, Seradigm; 1400-500), 2 mM L-glutamine (Corning, Corning, NY, USA; 25-005-CI), 100 IU of penicillin-streptomycin (Corning; 30-002-CI), and 100 μg/mL:0.25 μg/mL antibiotic/antimycotic (Corning; 30-002-CI). #1 cells have been previously described [11]. For transfection, Jetprime (Polyplus New York, NY, USA; 114-15) or Lipofectamine 3000 (Thermo Scientific, Waltham, MA, USA) transfection reagents were used following the manufacturers’ instructions. At 20–24 h post seeding, biotin was added to each dish to a final concentration of 50 μM and incubated for an additional 24 h before harvesting the cell lysates. Generation of the HEK293 CD63 CRISPR cell line has been previously described [18,19]. 

### 2.2. Plasmids

Generation of BioID constructs have described previously [20]. CD63 was amplified from RFP-CD63 pQCXP CMV/TO vector (for CD63 BirA*) or from pCT-CD63-GFP (for BirA*CD63). For CD63 BirA*, Nhe and EcoRI restriction fragments were ligated into pcDNA3.1-MCS BioID by overnight incubation with T4 DNA ligase. In the case of BirA*CD63, EcoRI and HindIII restriction enzymes were used for the digestion, and the fragments were inserted into pcDNA3.1-myc-BioID as above. The ligations were transformed into DH5α and plated on ampicillin-resistant LB-agar plates, incubated overnight at 37 °C for isolation of transformed colonies. Point mutations were made on CD63 sequences in the cases of both CD63 BirA* and BirA*CD63 on the guide DNA sequences using the GENEART mutagenesis kit (Invitrogen; A13282). The primers used were as follows: BirA_CD63-H to M_1F: 5’GCCTGTGCAGTGGGATTGATCGCCATGGGTGTCGGGGCAC3’ and BirA_CD63-H to M_1R: 5’GTGCCCCGACACCCATGGCGATCAATCCCACTGCACAGGC3’. This mutation facilitated the re-introduction of CD63 constructs under the CD63 CRISPR background successfully. The other constructs used in this study, GFP-LMP1, LMP1 BirA*, and BirA* LMP1, were also described previously [20].

### 2.3. Immunofluorescence Assay

HEK293-CD63 CRISPR cells were plated on fibronectin-coated coverslips placed in a 6-well plate at a density of 150,000 per well. At 24 h post seeding, cells were transfected with BirA*CD63 or CD63-BirA* using 5 ug of DNA with or without GFP-LMP1, replacing with fresh media containing 50 uM Biotin. Twenty-four hours post-transfection, media were aspirated followed by washing the cells with PBS three times. The cells were fixed in 4% paraformaldehyde (PFA) for 10 min at room temperature. After washing three times with PBS, the cells were then blocked with 5% goat serum in PBS-T blocking buffer (phosphate buffered saline with 0.5% Tween20) for 30 min at room temperature. The cells were then incubated with anti-CD63 antibody (TS63, Abcam Cambridge; UK) and anti-LMP1 antibody (CS1–4, Dako, Agilent, Santa Clara, CA, USA) at a dilution of 1:150 in blocking buffer for 2 h. Cells were then washed three times in PBS and incubated with goat anti-mouse DyLight 594 (Thermo Scientific, R37121, Waltham, Mass., USA) and goat anti-rabbit 488 (Thermo Scientific, A11034) at a dilution of 1:1000 in blocking buffer for 1 h at room temperature in the dark. In case of streptavidin staining, streptavidin 488 (Thermo Scientific, 21832) at a dilution of 1:1000 in blocking buffer was used during secondary antibody incubation in a similar way as above. After incubation, secondary antibodies were removed, and the cells were washed three times with PBS. The cells were then counter stained with DAPI diluted in PBS (1:10,000) for 10 min at room temperature followed by washing with PBS three times. The coverslips were mounted on glass slides with a drop of mounting media (4% propyl gallate and 90% glycerol in PBS), and the slides were sealed with clear nail polish and allowed to dry overnight at in the dark at 4 °C. Confocal images were obtained with a 63X objective on a Zeiss LSM 880 microscope and processed using the Zen 2 (Blue edition, V2.0.0.0; Zeiss) software package. 

### 2.4. Cell Lysis and BioID Pull-down

Control cells or cells overexpressing CD63-BioID constructs with or without LMP1 were grown to confluency. After collecting supernatant for EV analysis, the cells were washed with PBS and BioID lysis buffer was added (50 mM tris, pH 7.6, 500 mM NaCl, 0.4% SDS, 5 mM EDTA, 2% Triton x-100, 1 mM DTT, and HALT protease inhibitor (Thermo Scientific, 78420B)). The cells were lysed on ice for 10 min, scraped into 1.5 mL tubes, and sonicated (Bioruptor, Diagenode, Denville, NJ, USA) for 8 bursts, 30 s each with 30 s cooling down in between. An equal volume of 50 mM Tris, pH 7.6, was added to bring the lysis buffer concentration to 1X. Sonication should be repeated until all viscous clumps are disappeared and the lysate looks clear. The lysate was then centrifuged at 4 °C for 15 min at 14,000 rpm to clear any insoluble materials.

For BioID pull-down, an equal quantity of protein from untransfected and cells transfected with CD63-BirA* or CD63-BirA* with GFP-LMP1 were incubated with streptavidin magnetic beads (Dynabeads MyOne C1; Life Technologies Carlsbad, CA, USA; 65002), at a rate of 100 μl per milligram total protein overnight at 4 °C. The next day, the beads were washed twice using BioID wash buffer 1 (2% SDS (*w/v*) in HPLC grade water), once each with BioID wash buffer 2 (0.1% (*w*/*v*) deoxycholic acid, 1% (*w*/*v*) Triton X-100, 1 mM EDTA, 500 mM NaCl and 50 mM HEPES (pH 7.5)) and buffer 3 (0.5% (*w*/*v*) deoxycholic acid, 0.5% (*w*/*v*) NP40, 1 mM EDTA, 250 mM LiCl and 10 mM Tris (pH 7.4)), and twice with wash buffer 4 (50 mM Tris pH 7.6, 50 mM NaCl). Finally, the samples were centrifuged at 5000 g for 3 min, and all the residual wash buffer was removed. A total of 2X Laemmli sample buffer containing 50 µm of biotin was added to the beads, boiled for 5 min, and loaded into the gel for mass spectrometry or Western blotting.

### 2.5. Immunoprecipitation

LCLs were enumerated and seeded in 150 mm dishes. Cells were harvested and centrifuged at 500× *g* for 2 min at 4C. The supernatant was removed, and the cells were washed with ice-cold PBS. The cell pellet was resuspended in Co-IP lysis buffer (20 mM Tris pH 7.6; 2 mM EDTA; 10% glycerol; 1% Brij98; 150 mM NaCl) (1 mL per 1E7 cells) on ice for 10 min. The cell lysate was centrifuged for 10 min at 13,000× *g* at 4C, and the supernatant was transferred to a fresh tube. The magnetic beads (Thermo MagnaBind Protein G #21349) were resuspended, and 20 µL of slurry was transferred to a new tube. The beads were placed on a magnetic rack, and the supernatant was removed/discarded. The beads were resuspended in 200 µL of lysis buffer and mixed by pipetting. Then, the beads were placed on the rack, the supernatant was discarded, and the process was repeated two more times. The CD63, LMP1 or Mouse IgG antibody (Invitrogen antiCD63 TS63 #0628D; Abcam LMP1 cs1–4 #ab78113/LMP1 S12 1:1; Millipore normal mouse IgG #12-371) was added to the cell lysate (5–10 µg ab to 500 µg/mL lysate) and the complexes were allowed to form in a rotator overnight at 4C. The lysate/antibody solution was transferred to the pre-washed beads and incubated in a rotator at RT for 30 min. The beads were placed on a magnetic rack, the supernatant was removed, and they were stored at −80C (in case further processing was required). The beads were washed with lysis buffer four times as previously described. The beads were resuspended in a strong lysis buffer (5% SDS, 10 mM EDTA, 8 mM Urea, 120 mM Tris HCL pH 6.8, 3% B-mercaptoethanol), and the protein was quantitated using the EZQ kit (Invitrogen, Carlsbad, CA, USA; #R33200). The CD63 pre-conjugated beads (Invitrogen #10606D) were added to the lysate directly and incubated overnight in the rotator at 4C. The flowthrough was removed and stored at −80C. The beads were washed and eluted as described previously.

### 2.6. Western Blots

To validate the biotinylated proteins, the samples were loaded and separated in 4–20% sodium dodecyl sulfate polyacrylamide gels (Lonza, 59111, Morristown, NJ, USA). The proteins were then transferred to a nitrocellulose membrane (GE Healthcare, 10600002). The membranes were blocked with 5% (weight/volume) fat-free milk powder in TBS-T either overnight at 4 °C or for one hour at room temperature. Membranes were then probed for proteins with vimentin (Santa Cruz Biotechnology, SC-6260,Dallas, TX, USA), STAT3 (Santa Cruz Biotechnology, SC-482), Syntenin-1 (Santa Cruz Biotechnology, SC-100336), TSG101 (Santa Cruz Biotechnology; SC-7964), HSC70 (Santa Cruz Biotechnology; SC-7298), CD63 (Santa Cruz Biotechnology; SC-15363), ALIX (Santa Cruz Biotechnology, SC-49268), BirA (GeneTex, GTX14002, Irvine, CA, USA), LMP1 (Santa Cruz Biotechnology, SC-57721) Integrinβ1 (Cell Signaling, 9699), Flotillin-2 (Santa Cruz Biotechnology, H-90), Rab8A (Santa Cruz Biotechnology, SC81909), Rab21A (Santa Cruz Biotechnology, SC81917), mTOR1 (Cell signaling, Danvers, MA, USA; 2983), Clathrin (Cell Signaling, 4796P), NEDD4L (Santa Cruz Biotechnology, SC514954), PP2A-alpha (Santa Cruz Biotechnology, SC56954), and PP2A-delta (Santa Cruz Biotechnology, SC81605). Secondary antibodies conjugated to horseradish peroxidase were added to appropriate blots after the primary antibody incubation. Enhanced chemiluminescent (ECL) HRP substrate was added for picogram (Thermo Scientific, #1856136) or femtogram (Amresco, Solon, OH, USA; 1B1583) protein detection thresholds. Chemiluminescence was detected using the LAS4000 luminescent image analyzer and software Version 8.1 of Image Quant-TL (GE Healthcare Life Sciences). To detect all biotinylated proteins in lysates, atreptavidin HRP (Thermo, 21130) was incubated over the nitrocellulose membrane as described above, except the solution was incubated for 40 min at room temperature, washed, and then incubated in substrate as above.

### 2.7. Mass Spectrometry 

Proteins pulled-down from an equal quantity of cell lysates were ran in a 4 to 20% polyacrylamide gel (59511; Lonza, Basel, Switzerland) for purification and separation by SDS-PAGE. Gels were fixed and stained with a Coomassie staining protocol as described previously [20,21]. Samples were fractionated, subjected to in-gel trypsin digestion, and collected peptides were dried in a speed vac. The peptides then submitted to the Florida State University Translational Science Laboratory for liquid chromatography–tandem mass spectrometry (LC-MS/MS) and analyzed as previously described [20]. Spectral counts were analyzed in Scaffold version 4.10, using a 99.0% protein threshold, and a minimum of 3 peptides were required for identification. Peptide identity was accepted if the Scaffold Local FDR algorithm demonstrated a probability greater that 95.0%.

### 2.8. Isolation of Extracellular Vesicles

An equal volume of cell culture supernatants was collected from HEK293 CD63 knock-out cells, untransfected or overexpressing CD63-BirA*h-m or BirA*CD63 h-m in the presence or absence of LMP1. The supernatants were then processed using the ExtraPEG method [22] or ultracentrifugation. Briefly, the collected supernatants were spun down at 500 g for 5 min followed by centrifugation at 2000× *g* for 10 min and 10,000 *g* for 30 min, respectively. For ExtraPEG, an equal volume of 2X PEG (16% polyethylene glycol and 1M sodium chloride) was added, mixed well, and incubated at 4 °C overnight. EVs were isolated by centrifuging at 3200× *g* for 60 min and further cleaned by resuspending in 1.0 mL filtered PBS, followed by ultracentrifugation at 54,000 rpm for 70 min. The EVs isolated were used for Western blotting or NTA analysis.

### 2.9. Protein Enrichment Analysis

The CD63 interacting proteins were compiled from three separate biological replicates for enrichment analysis. The Kyoto Encyclopedia of Genes and Genomes (KEGG) and biological process (GO) pathway analyses were conducted using ShinyGO v0.61 [23]. Cellular compartment enrichment analysis was performed using FunRich v3.1 [24]. Enrichment categories with *p* values < 0.05 were considered statistically significant

### 2.10. Statistical Analysis

Significance of the results was determined by Student’s two-sample *t*-test and ordinary one-way ANOVA. Figures were assembled by using Microsoft Excel, Adobe Photoshop CC2019, GraphPad 8.3, and CorelDraw 2019 software programs. The graphical abstract was generated using BioRender.

## 3. Results

### 3.1. Expression of CD63 BioID Constructs in CD63 Knock out Cells

**Figure 1:**
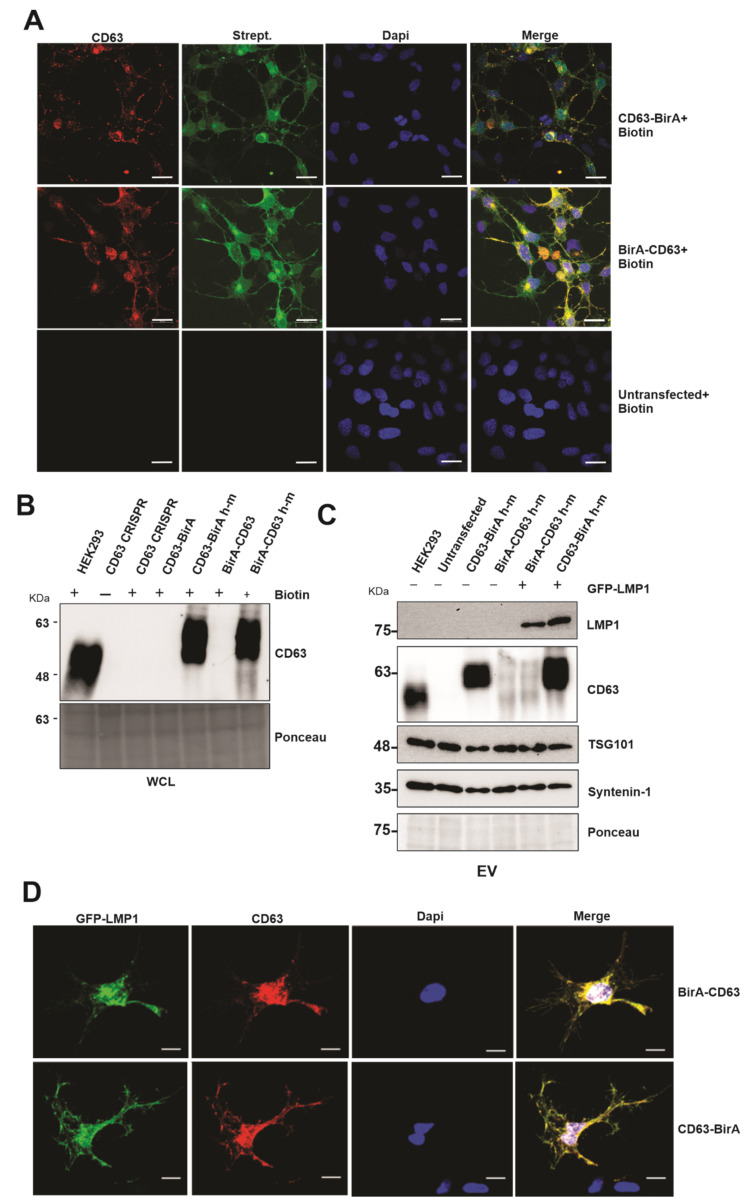
Expression of CD63-BioID constructs and validation of CD63-dependent exosome targeting of LMP1. CD63-BirA human constructs were mutated to mouse in the guide DNA sequence and expressed in HEK293 cells with CD63 CRISPR background. Human to mouse (h–m) mutations in the guide target sequence allowed successful translation of over-expressed constructs. (**A**) Co-localization of CD63 and biotinylated proteins: double immunofluorescence staining of CD63 and Alexa-488 conjugated streptavidin. Co-localization indicates CD63-BirA biotinylated proximal and interacting proteins. (**B**) Western blot analysis using anti-CD63 antibody showed successful expression of CD63 in HEK293 CRISPR cells. (**C**) EVs purified from the supernatant of CD63 CRISPR or cells expressing BirA-CD63 or CD63-BirA with or without GFP-LMP1 were purified using the ultra-centrifugation method and were used for Western blot. The results indicate that CD63 h-m mutant constructs can mediate exosome targeting of LMP1. (**D**) Immunofluorescence staining using anti-CD63 and anti-GFP antibodies using the cells expressing CD63 BioID h-m and GFP-LMP1. Co-localization indicates CD63 h-m mutants can interact with LMP1. WCL: whole cell lysate. Scale bar: 20 µm.

The use of BioID constructs for the identification of proximal interacting proteins has been previously described [20,25]. This approach takes advantage of the mutated bacterial protein biotin ligase that is fused to the protein of interest and expressed in the chosen cell lines. The mutation in the ligase abrogates its specificity towards natural substrate and is still able to biotinylate the proximal interacting proteins [26,27]. We over-expressed the CD63 BioID constructs in HEK293 cells expressing CD63 CRISPR to maximize protein identification and to avoid losing any identification due to the binding of endogenous CD63 and thereby limiting its availability to bind to BirA-tagged CD63. The CD63 BioID constructs reintroduced in the HEK293 cells had a mutated guide DNA sequence from human to mouse (h–m) on the human cDNA so that it is no longer targeted by Cas9. The CD63 constructs had fusion proteins (BirA) on either the N or C terminus (CD63-BirA*, BirA*-CD63). Immunofluorescent confocal microscopy analysis showed that the both CD63-BirA* and BirA*-CD63 constructs co-localized with the biotinylated proteins (streptavidin Alexafluor-488) (Figure 1A). To confirm that the CD63 BioID were functional, the constructs were transfected into HEK293 expressing CD63 CRISPR. Western blot analysis of the CD63 BioID constructs demonstrated that they were functional and were able to target LMP1 into the EVs when co-expressed (Figure 1B,C). C-terminal tagged CD63 showed enhanced LMP1 EV packaging compared with the N-terminal tagged. Similar results have previously been observed when the LMP1 C-terminal was tagged in comparison to the N-terminal region [20]. CD63 has been shown to associate with LMP1, and this interaction is important for LMP1 trafficking to EVs. Immunofluorescent confocal microscopy demonstrated that the CD63 BioID constructs co-localized with GFP-LMP1, showing that the mutant constructs are capable of interacting with LMP1, as previously described (Figure 1D) [18,28,29]. Image analysis of both BirA-CD63 and CD63-BirA showed spatial overlap with GFP-LMP1. Together, these results show that the CD63 BioID constructs are functional and can co-localize with and traffic LMP1 to EVs.

### 3.2. CD63 BioID Constructs Biotinylate Proteins

**Figure 2:**
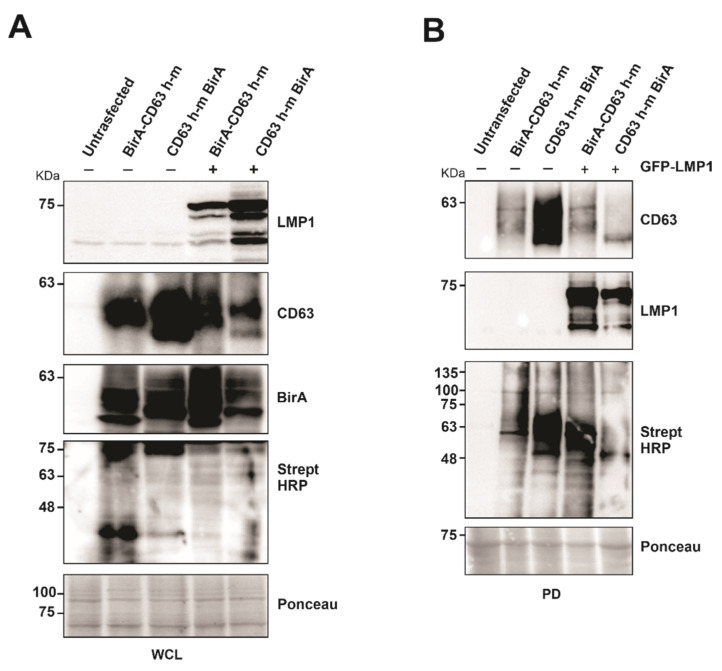
Expression of CD63-BirA constructs alone or in the presence of LMP1 exhibit biotinylase activity. (**A**) Protein expression and BirA activity were verified using indicated antibodies. (**B**) Biotinylated proteins were pulled-down from the total cell lysate using streptavidin magnetic beads. Pull-down efficiency was verified by Western blotting using the indicated antibodies. WCL: whole cell lysate; PD, pull-down.

To evaluate the capability of the CD63 BioID constructs to biotinylate the interacting proteins, BirA*-CD63 and CD63-BirA* were expressed in HEK293 CD63 CRISPR cells in the presence or absence of LMP1. Western blot analysis of whole cell lysate showed both the CD63 BioID constructs and LMP1 expressed in the cells. Expression of CD63 BioID constructs was also evident by the presence of BirA in the whole cell lysate (Figure 2A). Expression of CD63 BioID constructs and its efficiency in biotinylating proximal proteins was indicated by CD63, BirA, and streptavidin-HRP blots, respectively (Figure 2A,B). We consistently saw a lower expression of N-terminal BirA constructs compared to the C-terminal tagged counterparts. CD63 is highly glycosylated, causing multiple bands and smearing on immunoblots. Similarly, LMP1 is proteolytically cleaved, generating smaller molecular weight products. As determined previously, Strept-HRP blots are notoriously messy, likely due to the quality of the antibody and the large number of biotinylated peptides on the nitrocellulose [20]. Regardless, the blots in Figure 2 validate expression of the constructs and their ability to biotinylate proteins which was later confirmed by mass spectrometry. Subsequent pull-downs on the whole cell lysate were carried out using streptavidin coated magnetic beads to verify the presence of biotinylated proteins. The results showed the proteins biotinylated were efficiently pulled-down by the beads and visualized. The presence of CD63 in the pull-down indicates self-biotinylation and LMP1, as an interaction partner of CD63, as shown previously [20].

### 3.3. Identification of CD63 Interacting Proteins Using BioID Method

**Figure 3:**
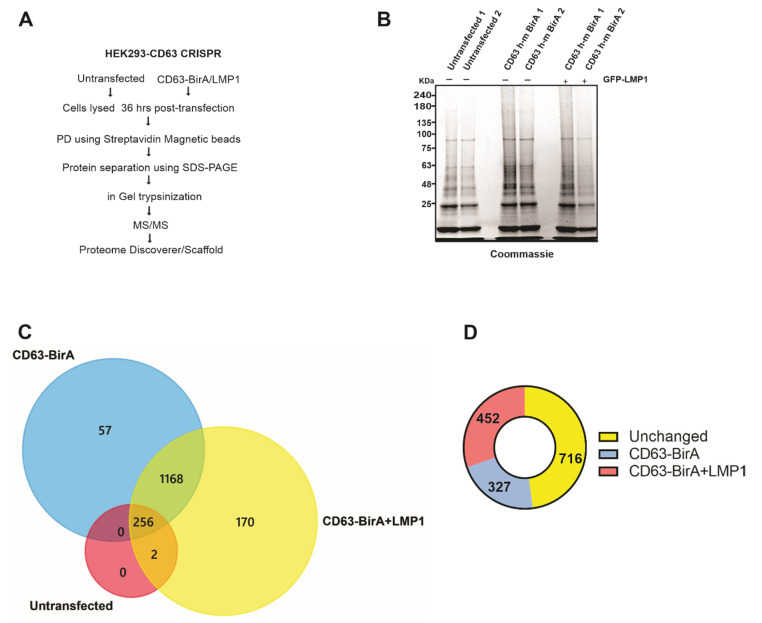
Affinity pull-down and mass spectrometry-based identification of associated proteins. (**A**) Workflow adopted to identify CD63 interacting proteins using the BioID approach coupled with mass spectrometry. (**B**) CD63-BirA constructs were over expressed with or without GFP-LMP1 in HEK293 CD63 CRISPR cells. Biotinylated proteins were pulled-down from the total cell lysate using streptavidin magnetic beads. Bound proteins were eluted using sample buffer + 50 mM biotin and resolved on a 4–20% gel, followed by staining with Coomassie brilliant blue. (**C**) Protein bands were destained, trypsinized, and identified using liquid chromatography coupled with mass spectrometry (LC-MS/MS). Mass spectrometry files were searched against human database using Proteome Discoverer software and further analyzed using Scaffold program. Venn diagram showing the proteins identified in untransfected, transfected with CD63-BirA, and with CD63- BirA and GFP-LMP1. (**D**) LMP1 modifies the CD63 interactome. The presence of LMP1 changes the interacting proteins to more strongly interacting or less strongly interacting.

For the identification of the CD63 interacting protein, we utilized the CD63-BirA construct because it had good expression and showed more biotinylated proteins on the Western blot compared to BirA-CD63. In order to identify the CD63 interactome, CD63-BirA was expressed in CD63 knocked-out HEK293 cells with or without GFP-LMP1, and the untransfected cells served as control. A schematic of the technique used for identification of the CD63 interacting proteins is given in Figure 3A. Streptavidin-coated magnetic beads were used to isolate the biotinylated proteins and separated using SDS-PAGE. The gel was subsequently Coomassie stained to visualize the protein bands before being fractionated based on band intensity and processed for mass spectrometry analysis (Figure 3B). 

We identified about 1600 total proteins (File S1), with roughly 1495 proteins unique to the CD63-BirA and CD63-BirA + LMP1 interacting protein groups (File S2) (Figure 3C). One hypothesis investigated was that LMP1 modifies CD63 direct and proximal interacting proteins. Co-expression of LMP1 along CD63-BirA changed the interactome considerably. About 170 protein were identified to be unique to CD63-BirA + LMP1, and about 57 proteins were unique to CD63-BirA alone. Total spectrum-based quantitation revealed about 327 proteins to be enriched two-fold or higher in the interactome when expressing CD63-BirA only and about 452 proteins enriched two-fold or stronger when LMP1 was co-expressed. Additionally, another larger pool consisting of 716 proteins remained unchanged (Figure 3D). 

**Figure 4:**
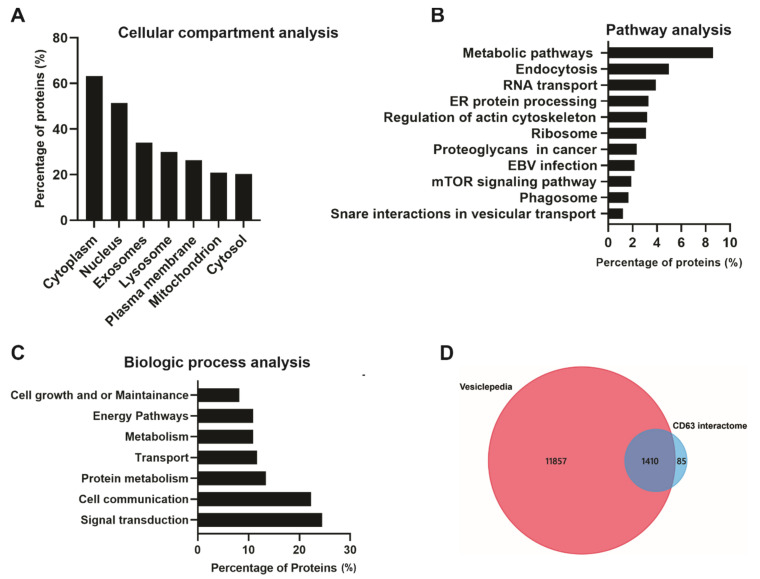
Bio-informatic analysis of CD63 interacting proteins identified using the Bio-ID approach. FunRich analysis: (**A**) Based on cellular compartment shows a higher enrichment in the cytoplasm and nucleus, exosome, and lysosomes. (**B**) Pathway analysis shows metabolic pathways, endocytosis, and RNA transport and protein processing. (**C**) Biological processes show the identified proteins are enriched signal transduction, cell communication, and protein metabolism. Vesiclepedia analysis: (**D**) The identified proteins were compared to the Vesiclepedia database and we found the majority of proteins are listed in the database.

A total of 1495 CD63 proximal interacting proteins were further subjected to bio-informatics analysis to understand the protein–protein interactome. Cellular compartment analysis showed the highest enrichment in the cytoplasm, nucleus, exosome, and lysosome (Figure 4A). This was not surprising as CD63 is a cytoplasmic protein highly localized in cellular membranes including the plasma membrane; therefore, the majority of the identified proteins belong to cytoplasm. CD63 has also been shown to be enriched in late endosomes and lysosomes leading to secretion into EVs. Pathway analysis showed that a large number of interacting proteins were involved in endocytosis, RNA transport, ER protein processing, ribosomes, EBV infection, proteoglycans in cancer, mTOR signaling and phagosomes (Figure 4B). Multiple studies have demonstrated the role of CD63 in endocytosis and intracellular trafficking of different proteins including major histocompatibility complex (MHC) class II from the plasma membrane to endosomes [30,31]. Some of the identified protein associated with endosomal system trafficking include CHMP4B, CHMP2B, CHMP5, CHMP6, HRS, EEA1, CAV1, VPS26A, VPS45, NEDD4L, and Rab GTPases (Rab10,11,31,5C,7A, and 11B). CD63 has also been implicated in the regulation of mTOR signaling activation by LMP1 [19]. The biological processes analysis for the dataset revealed enrichment in signal transduction, cell communication, protein metabolism, transport, energy pathways and, cell growth, and maintenance (Figure 4C). CD63 has been found to be a negative regulator of mitogen-activated protein kinase (MAPK)/ERK and noncanonical NF-κB pathway activation in the context of LMP1 [18,28]. Data from these studies suggest that CD63 is responsible for transporting different signaling molecules to the extracellular environment or recipient cells. Comparison of the dataset to the Vesiclepedia database showed most of our identified proteins have been previously described in vesicles (Figure 4D). 

To further understand how LMP1 modifies CD63 direct and proximal interacting proteins, a comparison bioinformatic analysis of the proteins which were two-fold or higher as compared to CD63-BirA or CD63-BirA + LMP1 was carried out. Pathway analysis of the CD63-BirA-only dataset revealed enrichment in endocytosis, axon guidance, regulation of the actin cytoskeleton, SNARE interactions in vesicular transport, and phagosomes (Table 1, File S4). Most of the proteins enriched in endocytosis, SNARE proteins, and phagosome interaction pathways are not a surprise as CD63 likely interacts with these proteins during trafficking intracellularly through the endosomal pathway. Gene ontology (GO) biological process assessment of the CD63-BirA identified proteins showed enrichment mainly in protein/macromolecule localization and endosomal/vesicle-mediated transportation (Table 1, File S3). The majority of the proteins identified include Rab GTPases (Rab1A/B,5C,7A,8A/B,9A,21,23, 35, ARF6) and sorting nexins proteins (SNX1-3,5,6,9,11,12,17,27,30, VAMP3,4,7, and 8) which contribute to protein localization and cellular trafficking processes and are more likely to interact with CD63. The introduction of LMP1 into the CD63-BirA interactome modified the interacting proteins. Pathway analysis of the CD63-BirA + LMP1 proteins was enriched in RNA transport, proteasome, EBV infection, ER protein processing, and metabolic pathways (Table 1, File S6). Analysis of the GO biological processes demonstrated the identified proteins were mainly involved in metabolic processes and translation (Table 1, File S5). LMP1 has been shown to regulate and induce metabolic changes in cells which might lead to cell growth. These enriched pathways and biologic process seen when LMP1 is introduced are similar to what was identified and described using LMP1 BioID previously [20]. Collectively, these data show that the addition of LMP1 to the CD63 interactome modifies both direct and proximal interacting proteins, providing insights on the cellular processes that LMP1 manipulates.

### 3.4. Verification of CD63 Interacting Proteins

**Figure 5:**
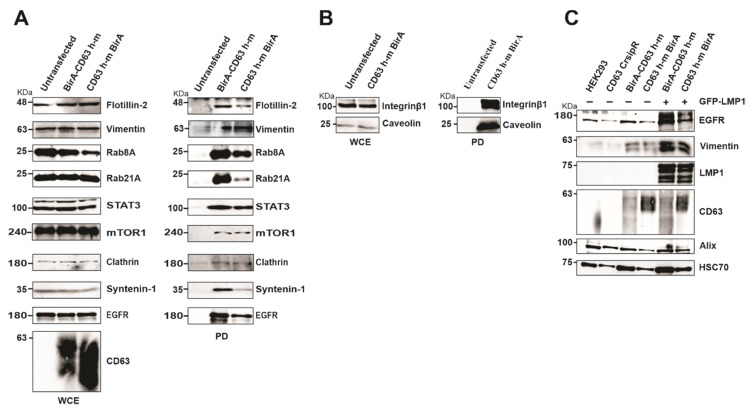
Verification of mass spectrometry results using Western blot and identification of EGFR and vimentin as CD63/LMP1 dependent exosome targeting proteins. (**A**) HEK 293 CRISPR cells were transfected with BirA-CD63 or CD63-BirA. Untransfected cells maintained as control. CD63 interacting proteins were pulled-down using streptavidin magnetic beads and subjected to Western blotting. (**B**) Only CD63-BirA expressing cell lysate were used for pull-down and Western blot. (**C**) Over-expression of CD63 alone or doubly with GFP-LMP1 leads to increased exosome targeting of vimentin and EGFR. Exosomes were purified using ultra-centrifugation method and lysed in sample buffer. Equal volume of sample resolved on a 10% SDS-PAGE and blotted against EGFR and Vimentin. The results show CD63 enhances exosome targeting of these proteins and is further enhanced in presence of LMP1. WCE, whole cell extract; PD, pull-down.

To verify the proteins identified in the CD63 interactome, HEK293 cells expressing CD63-CRISPR cells were over expressed with BirA-CD63 and CD63-BirA, and interacting proteins were pulled-down using streptavidin magnetic beads to visualize the biotinylated proteins. The pull-downs confirmed CD63 interaction with Rab GTPases and other proteins involved in the endolysosomal pathway including Rab8A and Rab21A, clathrin, syntenin-1, caveolin and flotillin-2 (Figure 5A). Interestingly, many of these proteins were previously found to be upregulated in EVs from EBV infected lymphocytes correlating with LMP1 expression levels in the EV producing cell lines [11]. The CD63 and syntenin-1 interaction has already been shown to be important for EV cargo packaging and release. CD63 also interacted with some signaling components including STAT3, mTOR1, EGFR and integrin Beta 1 (Figure 5A,B). Both EGFR and vimentin were previously implicated in LMP1 mediated signaling [32,33]. We investigated the role of CD63 in LMP1 dependent EV targeting of EGFR and vimentin. CD63 CRISPR cells were transfected with CD63-BirA or BirA-CD63 in presence or absence of LMP1 while untransfected cells and HEK293 wild type cells were kept as controls. Western blot analysis revealed that over-expression of CD63 leads to targeting EGFR and Vimentin to EVs (Figure 5C). Taken together these data give a strong indication that the identified proteins represent CD63 interactome comprising proximal, direct and indirect interacting proteins. 

### 3.5. CD63 Regulates LMP1 Interaction with mTor, Nedd4L and PP2A

**Figure 6:**
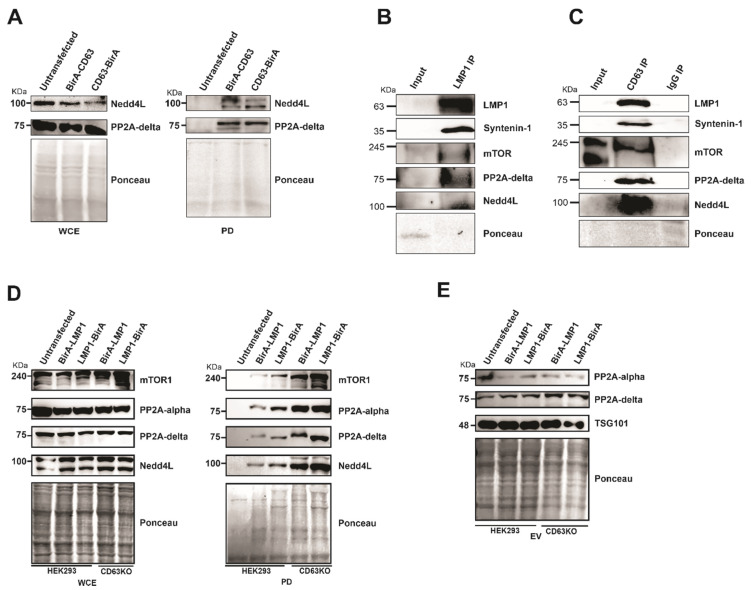
The absence of CD63 leads to increased interaction of mTOR, Nedd4L, PP2A. (**A**) Both Nedd4L and PP2A delta interact with CD63. Pull-down assays using CD63 BioID fusion proteins Figure 2. A delta with CD63. (**B**,**C**) Immunoprecipitation of mTOR, Nedd4L, and PP2A in #1 B-cells. Immunoprecipitation in the #1 cells was carried out using LMP1 antibodies and CD63 pre-conjugated beads followed by immunoblotting. (**D**) Pull-down experiments using LMP1 BioID fusion proteins followed by Western blotting using indicated antibodies demonstrates that LMP1 BioID fusion proteins pulled-down an increased level of the interacting proteins in the absence of CD63. At the same time, WCE shows a similar level of proteins in all the lanes. (**E**) The increased interaction with LMP1 in absence of CD63 is not due to differential targeting of the extracellular vesicles (EVs). EVs purified from supernatant were resolved on SDS-PAGE and blotted for the antibodies indicated. The result indicates that there is no obvious difference in EV targeting due to the absence of CD63.

Some of the CD63 interacting proteins identified include mTOR, ubiquitin ligase Nedd4L, protein phosphatase 2A alpha (PP2A alpha), and protein phosphatase 2A delta (PP2A delta). mTOR has been implicated in the formation and modulation of endophagosomes, which is CD63 dependent [19]. Furthermore, our previous studies demonstrated that CD63 regulate LMP1-dependent phosphorylation of mTOR at S2448 [19]. The interaction of CD63 with Nedd4L and PP2A delta was verified using Western blots in pulldown assays (Figure 6A). Furthermore, to verify the relevance of these interactions, we performed immunoprecipitation analyses in #1 B cells (LCLs) which are transformed by EBV and express LMP1. Immunoprecipitation using LMP1 antibodies or CD63 pre-conjugated beads showed interaction with mTOR, Nedd4L, and PP2A delta (Figure 6B,C). Both E3 ligase NEDD4L and PP2A has been also shown to regulate mTOR signaling pathway [34,35]. To assess the role of CD63 in modulating mTOR phosphorylation, we analyzed if mTOR can form a multiprotein complex with LMP1 and CD63. LMP1 BioID constructs were expressed in HEK 293 wild-type cells or cells expressing the CD63 CRISPR. A pulldown assay using streptavidin magnetic beads and immunoblotting analysis revealed interaction between LMP1 and mTOR (Figure 6D). Surprisingly, in the absence of CD63, an enhanced interaction between LMP1 and mTOR was observed. These data suggest that CD63-dependent down-regulation of mTOR at S2448 may be achieved through the decreased interaction of LMP1 and mTOR. Additionally, CD63 also affected LMP1 interaction with Nedd4L, PP2A delta, and PP2A alpha (Figure 6D).

It was shown that in presence of CD63, increased levels of mTOR were targeted into extracellular vesicles. This CD63-dependent targeting was enhanced when LMP1 was co-expressed, indicating the formation of a multiprotein complex and enhanced EV targeting leading to decreased levels of active mTOR in the cells. Adding to these, we also checked if PP2A showed a similar trend in targeting EVs in the absence of CD63. However, we observed only a minimal increase in targeting of both forms of PP2A in the absence of CD63 or in the presence of LMP1 (Figure 6E). Taken together, these data further support that CD63 is a regulator of LMP1 mTOR signaling and EV cargo manipulation.

## 4. Discussion

Tetraspanin CD63 is widely expressed in cells and commonly localizes into late endosomes, lysosomes, and the plasma membrane. The assembly and association with TEMs on the cell surface allows CD63 to interact with a plethora of proteins, which are involved in directing its cellular functions [36,37]. CD63 has long been used as a marker of EVs, and recently it has received much attention because of its role in EV biogenesis and sorting of different cargo. In this study we evaluated the broad CD63 interactome, identifying the direct and indirect binding proteins and the enriched biological processes these proteins are involved in. Our results show that the viral oncoprotein LMP1 modifies the CD63 interactome. The presence of LMP1 shifted the enrichment of biological processes of the biotinylated proteins to mainly metabolism from transportation in the presence of CD63 alone. This comprehensive analysis of CD63 proximal interacting proteins provides an insight into the different proteins required in protein trafficking, EV cargo sorting, and secretion.

CD63 direct or indirectly interacting proteins identified were shown to be involved in endocytosis. From the plasma membrane, CD63 has been shown to endocytose via the clathrin-coated vesicles, or alternatively, it is internalized through the caveolae-mediated endocytosis [38,39]. Proteins associated in these processes including caveolin-1 and the clathrin complex (PICALM, CLINT1, CLTC) were identified in the mass spectrometry dataset. The interaction between CD63 and caveolin or clathrin was further verified through affinity purification. CD63 harbors a tyrosine-based sorting motif which facilitates the recruitment of the clathrin coats and the binding of adaptor proteins [40]. These adaptor proteins (AP), AP-1, AP-2, AP-3, and AP-4, have been shown to interact with the sorting motifs to mediate transmembrane proteins such as the CD63 targeting of lysosomes [39,41]. Here we identified the AP-1 complex subunit beta, AP-2 complex subunit mu, and AP-3 complex subunit beta-1 as part of the CD63 interactome. AP-2 has been shown to mediate CD63 endocytosis through clathrin-coated vesicles from the cell surface to lysosomes, while AP-3 targets CD63 from recycling endosomes to lysosomes. Knockdown of AP-1 has been shown not to have any effect on CD63 trafficking of lysosomes [39,41,42]. Pols et al., even reported that no data have been shown to support that CD63 directly interacts with AP-1 [39]. Our data indicate that CD63 interacts with AP-1 either directly or indirectly. More studies seek to understand the role of the AP-1 complex in CD63 endocytosis. Taken together, these data show that CD63 likely interacts with proteins mediating the internalization of different molecules on the cell surface and transfer the cargo into the endocytic or secretory pathway. 

The sorting of CD63 in intraluminal vesicles (ILVs) is facilitated by the endosomal sorting complex required for transport (ESCRT) machinery. The CD63 interacting proteins present in our dataset which are involved in the ESCRT pathway include HRS, CHMP4B, CHMP2B, CHMP5, CHMP6, EEA1, CAV1, VPS4A, VPS26A, VPS45, and NEDD4L. Both ESCRT-dependent and ESCRT-independent cellular machineries such as CD63 have been demonstrated to drive biogenesis of EVs and cargo sorting [43]. Proteomic analyses have shown that EVs derived from B cells, tetraspanins such as CD63, CD82, and CD81, are 100-fold enriched as compared to the transferrin receptor [44]. Knockdown of ESCRT components Alix, HRS, and CHMP4A/B/C has been shown to decrease the number of EVs secreted and the amount of CD63 sorted in these EVs [45,46,47]. The association of CD63 and TEMs allows tetraspanin proteins such as CD63 to act as EV cargo sorting and budding. CD63 has been demonstrated to guide the sorting of melanosomal protein PMLE (amyloidogenic pigment cell-specific type I integral membrane protein) into ILVs in the ESCRT-independent pathway [48]. CD63 also mediates the sorting and secretion of LMP1 into EVs [18,28]. Using the BioID method, we have previously identified different ESCRT pathway components as LMP1 interacting proteins that have shown to be involved in EV sorting and formation [20]. These data suggest that both LMP1 and CD63 utilize the ESCRT pathways and associated proteins for trafficking and sorting into late endosomes. The detailed mechanisms by which these proteins are recruited or enter the ESCRT pathway are unknown; it may be that CD63 alone recruits LMP1 to the endosomal membrane or uses the help of other adaptor proteins such as Syntenin-1, or alternatively, it might be that the whole CD63-LMP1 complex is recruited at the same time to the endosomal membranes from the plasma membrane to enter the ESCRT pathway. More studies should be carried out to determine the contributions of the different ESCRT components in CD63 or CD63-LMP1 endocytic trafficking. Collectively, these data uncover the different components interacting with CD63 in the endosomal membrane and have major implications on EV cargo sorting and manipulation. 

Rab proteins are important mediators of vesicle formation and intracellular vesicle trafficking between different subcellular compartments. Our bioinformatic analysis of the CD63 interactome showed an enrichment of SNARE proteins and Rab GTPases. Rab GTPases proteins identified in the CD63 BioID interactome include Rab1A/B, 2A, 6A, 7A, 8A/B, 9A, 10, 11B, 18, 21, 22A, 23, 24, 29, 31, 35, and ARF6. Knockdown of Rab2B, 5A, and 9A in HeLa cells expressing MHC class II molecules have been shown to reduce EV secretion including CD63 and MHC class II packed in these vesicles [49,50]. Rab11 and Rab35 have been shown to be important in controlling the recycling of membrane components from the endosomal side to the plasma membrane, and this facilitate cellular processes such as cytokinesis [51]. Another Rab GTPase present in the CD63 interactome dataset is ARF6 which interacts with Syntenin-1 and regulates ILV budding and EV production. The depletion of ARF6 with siRNAs significantly decreased EV secretion and packaged proteins such as CD63, Syntenin-1, and Alix [45]. Mechanistically, ARF6 is required for CD63 ILV budding into MVBs hence CD63 accumulation in late endosomes upon ARF knockdown [45]. CD63 has already been shown to interact directly with Sytenin-1 which is thought to regulate the CD63-ALIX-Syntenin-1 complex interaction [20,52,53]. Taken together, these data reveal the wide network of CD63 with other important mediators in vesicle trafficking and EV secretion.

CD63 plays important role in LMP1 trafficking to EVs, and this also affects LMP1-mediated intracellular signaling [18,28]. The depletion of CD63 in cells limits LMP1-induced MAPK/ERK, NF-κB, and mTOR activation [18,19,28]. In the context of viral infection or viral-associated cancers, these findings have implications in understanding mechanisms of protein trafficking and signal transductions. Our data demonstrated that the introduction of LMP1 into the cells modifies the broader CD63 interactome. Enrichment analysis of the CD63-BirA + LMP1 biotinylated proteins revealed significant involvement in metabolic pathways and translation, while the CD63-BirA-only interactome proteins were highly involved in protein localization and transportation. Furthermore, the CD63-BirA + LMP1 interactome was enriched in proteins involved in signaling transduction, including CDK2, CALR, EZR, EGFR, TBK1, IRAK, MAPK1, MAPK3, MAP2K1, MAP2K2, MAP2K3, VIM, and STAT1. We have previously shown that most of these identified proteins are part of the LMP1 proximal interacting protein and validated some of the proteins’ interactions with LMP1 [20]. Modification of the CD63 network of binding proteins suggests that the signaling molecules upregulated by LMP1 might be using or interacting with CD63 to enter the endocytic pathway for EV secretion or proteasomal degradation. LMP1 might increase the recruitment of these key signaling components to lipid rafts where they can interact with CD63 for the trafficking through the endolysosomal system [32]. The sorting of LMP1 into EVs is thought to allow circumvention of proteasomal or lysosomal degradation [28,54]. LMP1 is ubiquitinated before degradation by the proteasome [55]. Here, we found that the introduction of LMP1 into the CD63 interactome also upregulated ubiquitin and proteasome-related components. Our results show that LMP1 alters the CD63 interacting proteins to enter the endocytic pathways, and this might lead to the manipulation of the EV content and cargo. Together, CD63 plays an important role in cellular trafficking of different proteins, EV cargo sorting, and vesicle formation. 

We have previously reported a LMP1-CD63-mTOR signaling axis in host cell growth and metabolism and the suppression of cellular autophagy facilitating virus latency and survival [19]. Indeed, many studies have shown varying roles of mTOR in the regulation of autophagy induced by various stressors involving hypoxia, infection, growth factor deficiency, and nutrient deficiency [56]. LMP1 activates mTOR signaling, and our results demonstrated that CD63 negatively regulates LMP1-dependent activation of mTOR, probably through increased secretion in to EVs [19,57]. Our experiments identified direct and proximal CD63 interacting proteins which are part of the mTOR pathway including mTOR, RICTOR, LAMTOR1, Nedd4L, PP2A alpha, PP2A delta, and PP2A B55 alpha. The results showed that mTOR, Nedd4L, PP2A alpha, and PP2A delta interact with LMP1, and the affinity of the interaction increases in absence of CD63, probably through decreased targeting of EVs thereby increasing cellular availability. In addition to mTOR, both phosphatases and ubiquitin ligase also showed increased interaction with LMP1 in the absence of CD63 but failed to show decreased EV targeting.

The cellular dynamics related to autophagy are tightly controlled by phosphorylation and ubiquitination. We have previously shown that mTOR is phosphorylated at serine residues in the presence of LMP1 [19]. During autophagy, the levels and activation of ULK1 kinase, which is upstream of autophagy, are controlled by ubiquitination and degradation by Nedd4L and dephosphorylation due to the physical detachment of mTOR from the complex [58,59,60]. mTOR binds to ULK1 directly utilizing its RAPTOR subunit, which is independent of ULK1 activation but dependent on nutrient availability [61,62]. PP2A is a serine threonine phosphatase which was shown to dephosphorylate p70S6Kinase, a downstream component in the mTOR pathway [59]. Another substrate of PP2A is ULK1, in which PP2A dephosphorylates at S638, and this phosphorylation is required for autophagy induction [63,64]. Another phosphatase, PP2A B55alpha, was also identified in the mass spectrometry study and is known to play an important role in the autophagy process by modulating phosphorylation of beclin 1 at S90 (BECN1) [65]. Further studies are required to identify the specific target of Nedd4L and PP2A, which are shown to interact with LMP1 more strongly in the absence of CD63 in modulating autophagy functions. Taken together, these data provide new insights, revealing that CD63 recruits a wide variety of signaling proteins into the LMP1 complex, directly or indirectly, thereby modulating multiple cellular functions including autophagy. Verification of the identified proteins using HEK293 only is a limitation of our study; therefore, future work will focus on the identification and verification of the LMP1 interactome in B-cells to increase the relevance of the results regarding EBV-associated cancers. 

## 5. Conclusions

Using the BioID method combined with mass spectrometry, we identified direct and proximal interacting proteins for tetraspanin CD63. This approach yielded about 1495 total CD63 interacting proteins, with about 327 proteins enriched two-fold or higher in the interactome when expressing CD63-BirA only and about 452 proteins enriched two-fold or higher when LMP1 was co-expressed. Several of the identified proteins in the interactome including adaptor proteins (AP-1, AP-2, AP-3), ESCRT pathway proteins, Rab GTPases, SNARE proteins and sorting nexins have been shown to have a major role in protein trafficking, EV cargo sorting, and secretion. LMP1 was found to alter the CD63 interactome through what may be a transfer of signaling molecules in the endocytic system for secretion as EVs. Interestingly, we found that LMP1 interacts with mTOR, Nedd4L, and PP2A, indicating the formation of a multiprotein complex with CD63, which potentially regulates LMP1-dependent mTOR signaling. This in-depth analysis of the CD63 interactome provide insights regarding possible therapeutic targets or biomarkers for EBV-associated cancers. 

## Figures and Tables

**Table 1:** Bio-informatic analysis of the proteins which were two-fold or higher as compared to the CD63-BirA or CD63-BirA + LMP1 dataset. Some of the top identified KEGG pathways and GO BP enrichment are shown in the table.

**CD63-BirA KEGG Pathway Enrichment**	**CD63-BirA + LMP1 KEGG Pathway Enrichment**
Endocytosis	Metabolic pathways
Axon guidance	RNA transport
Regulation of actin cytoskeleton	Spliceosome
Human cytomegalovirus infection	Epstein-Barr virus infection
Ras signaling pathway	Viral carcinogenesis
Phospholipase D signaling pathway	Proteasome
Tight junction	Protein processing in endoplasmic reticulum
**CD63-BirA gene ontology (GO) biological process enrichment**	**CD63-BirA + LMP1 gene ontology (GO) biological process enrichment**
Macromolecule localization	Organonitrogen compound biosynthetic process
Cellular localization	Protein-containing complex subunit organization
Protein localization	Cellular response to stress
Regulation of localization	Cellular amide metabolic process
Establishment of localization in cell	Peptide metabolic process
Regulation of cellular component organization	Amide biosynthetic process
Vesicle-mediated transport	MRNA metabolic process

## Supplementary Materials

The following are available online at https://www.mdpi.com/article/10.3390/v13040675/s1, File S1: Total identified proteins, File S2: CD63 interacting proteins, File S3: GO BP enrichment CD63_BirA, File S4: KEGG enrichment CD63_BirA, Files S5: GO BP enrichment CD63_BirA+LMP1, Files S6: KEGG enrichment CD63_BirA+LMP1.
