# Supplementary material for: Epstein-Barr Virus LMP1 Modulates the CD63 Interactome
Source: Viruses. 2021 Apr 15;13(4):675. doi: 10.3390/v13040675 (PMC8071190; doi:10.3390/v13040675)
Supplement: Supplementary file 1 [file viruses-13-00675-s001.zip › Uncropped blots_CD63 BioID_3_24_2021.pdf]

Figure 1

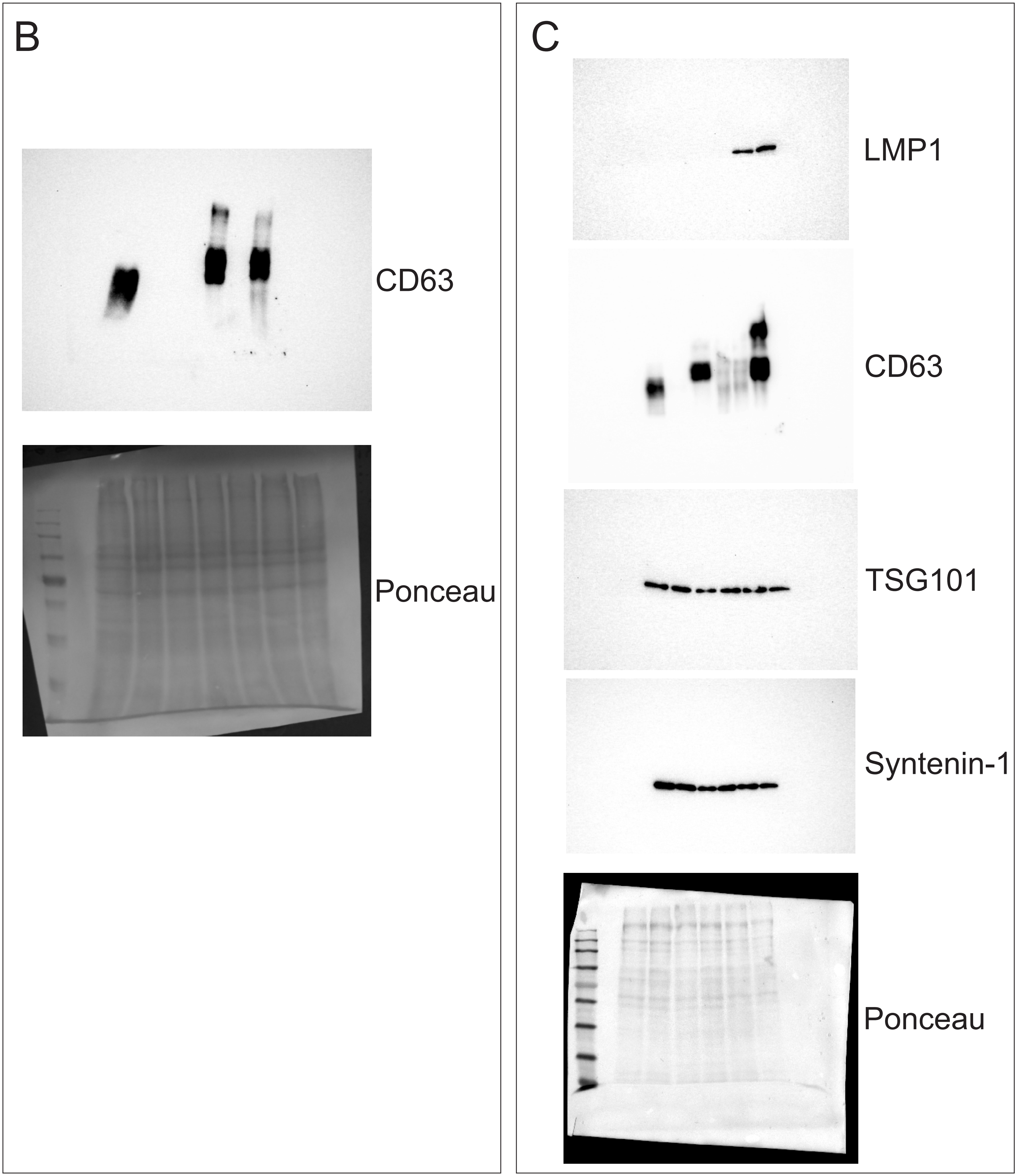

Figure 2

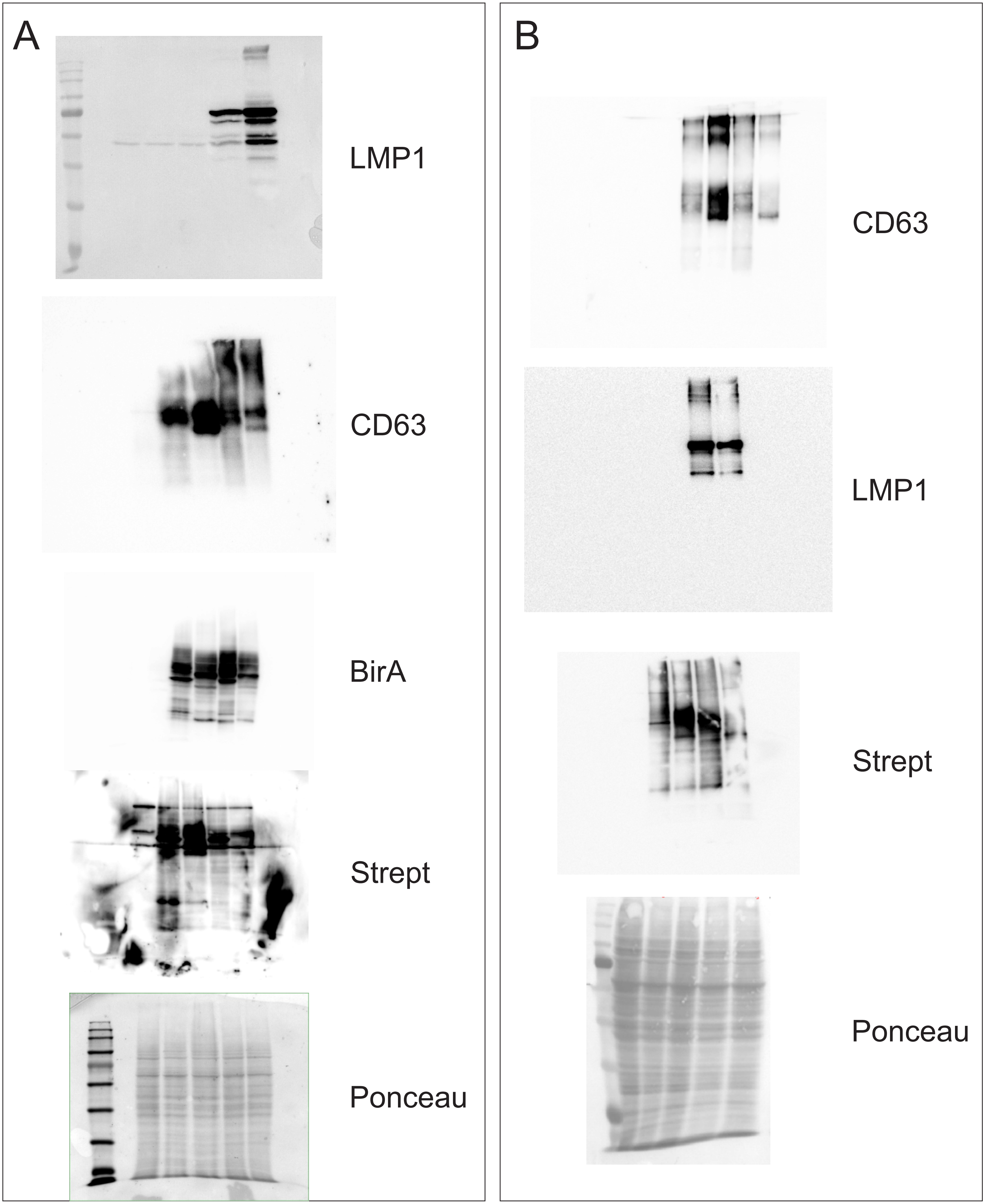

Figure 5A

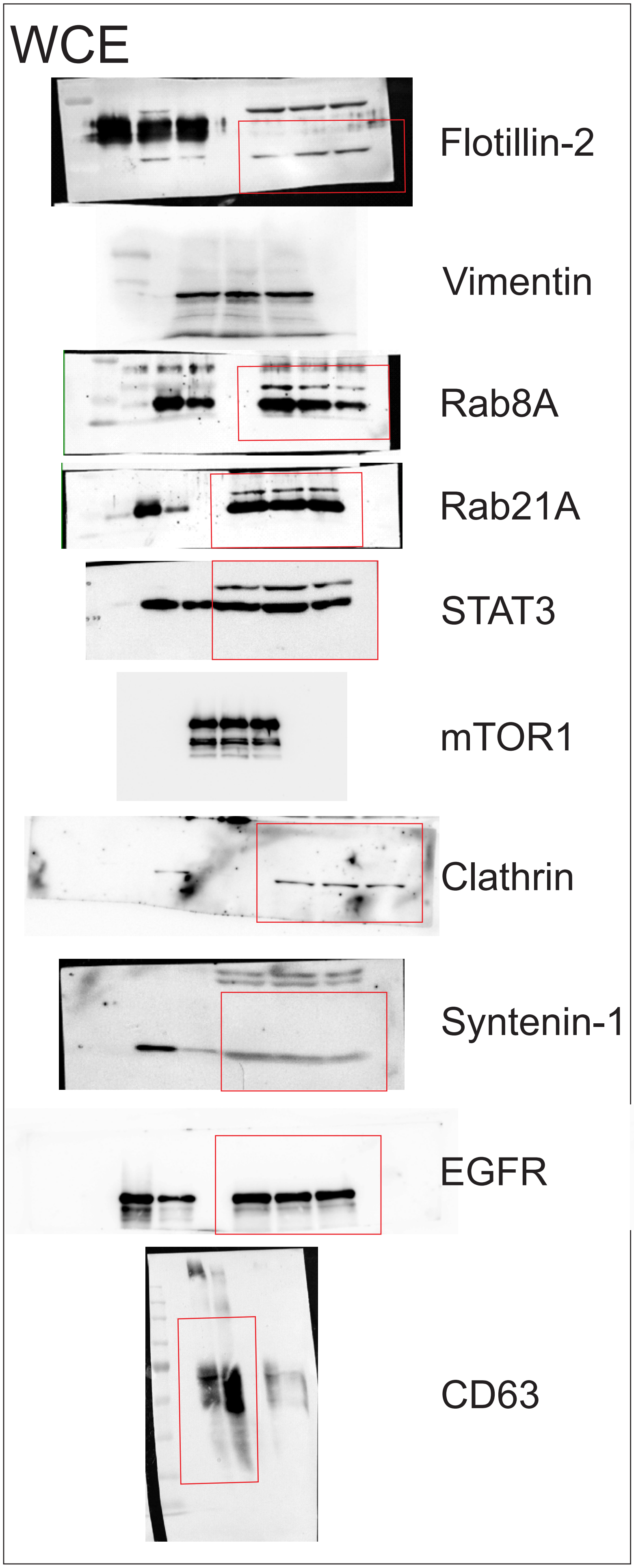
WCE
Flotillin-2
Vimentin
Rab8A
Rab21A
STAT3
mTOR1
Clathrin
Syntenin-1
EGFR
CD63

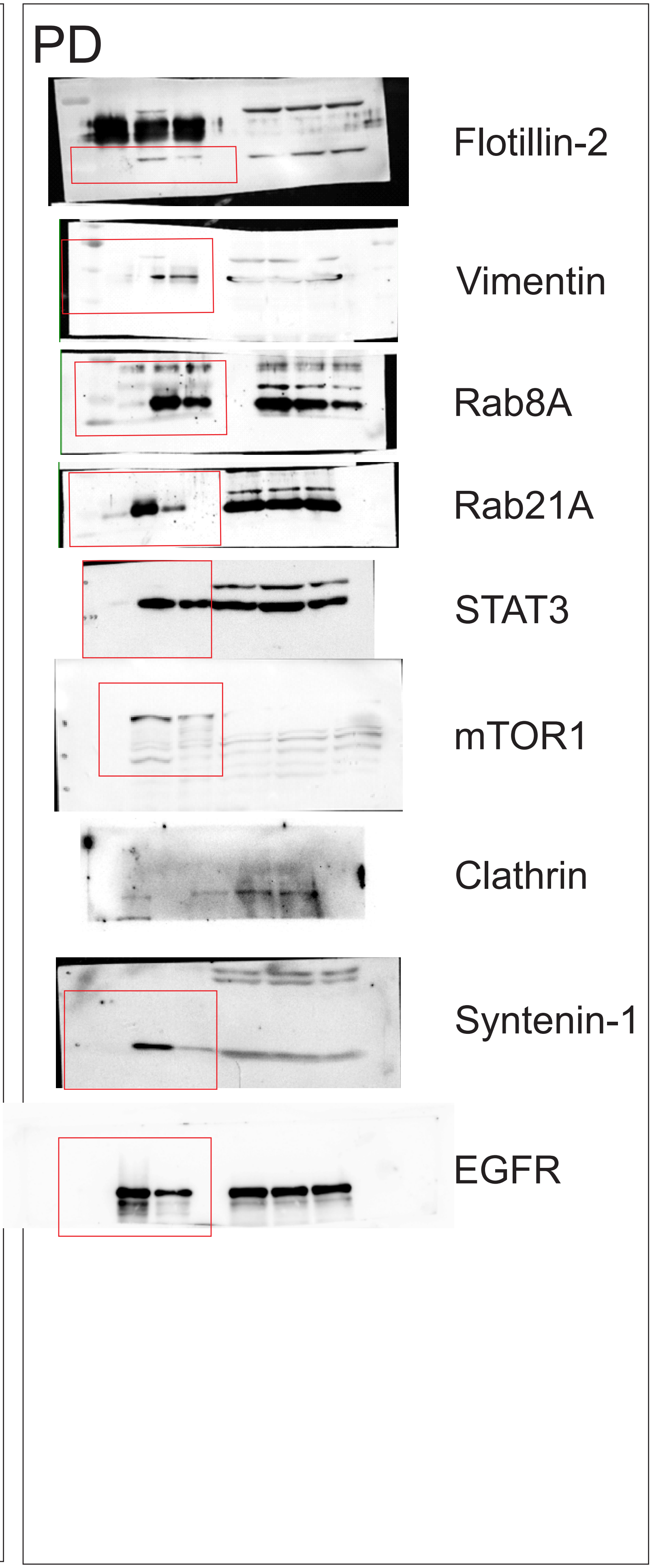
PD
Flotillin-2
Vimentin
Rab8A
Rab21A
STAT3
mTOR1
Clathrin
Syntenin-1
EGFR

Figure 5

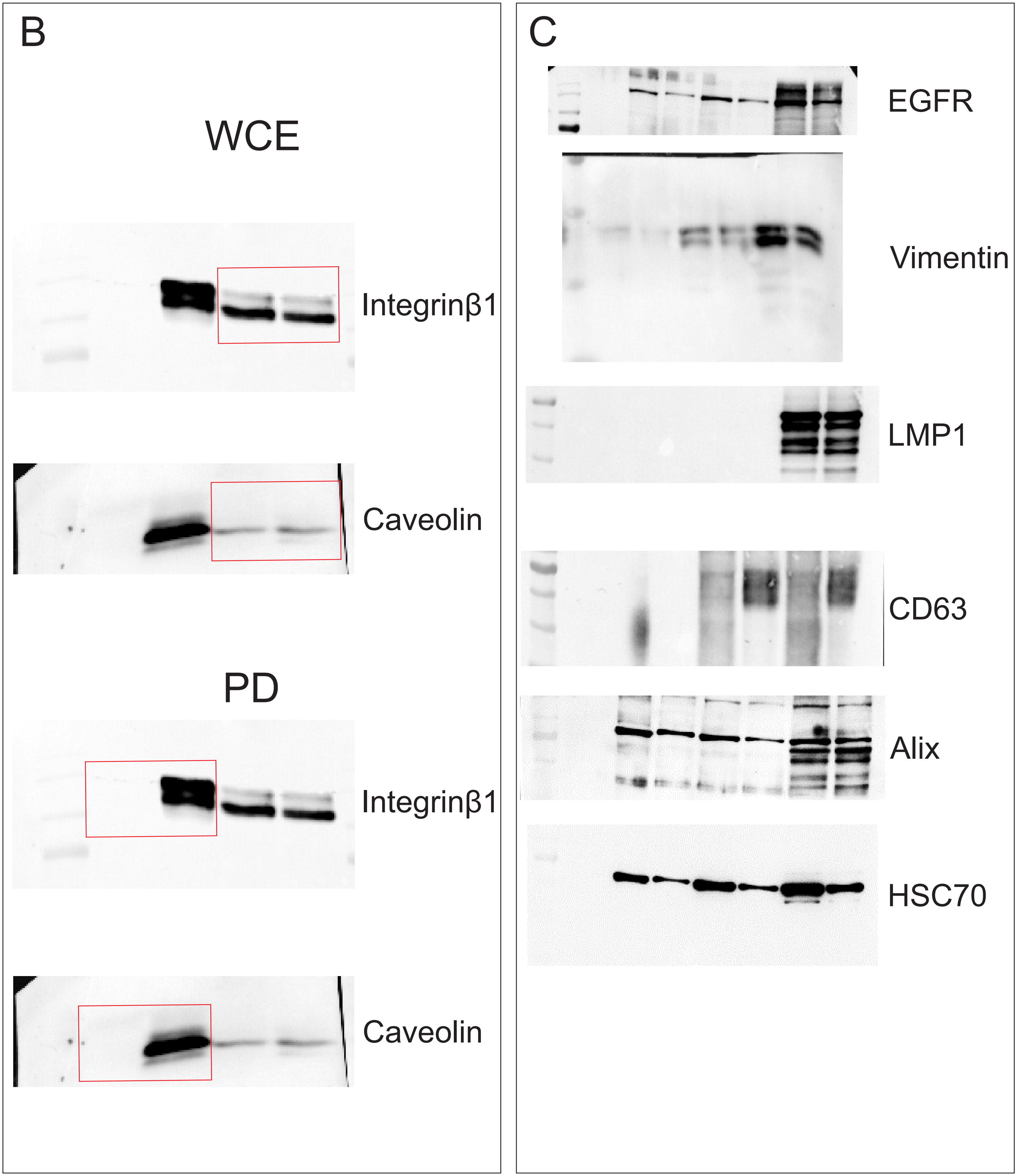
B
WCE
Integrinβ1
Caveolin
PD
Integrinβ1
Caveolin
C
EGFR
Vimentin
LMP1
CD63
Alix
HSC70

Figure 6

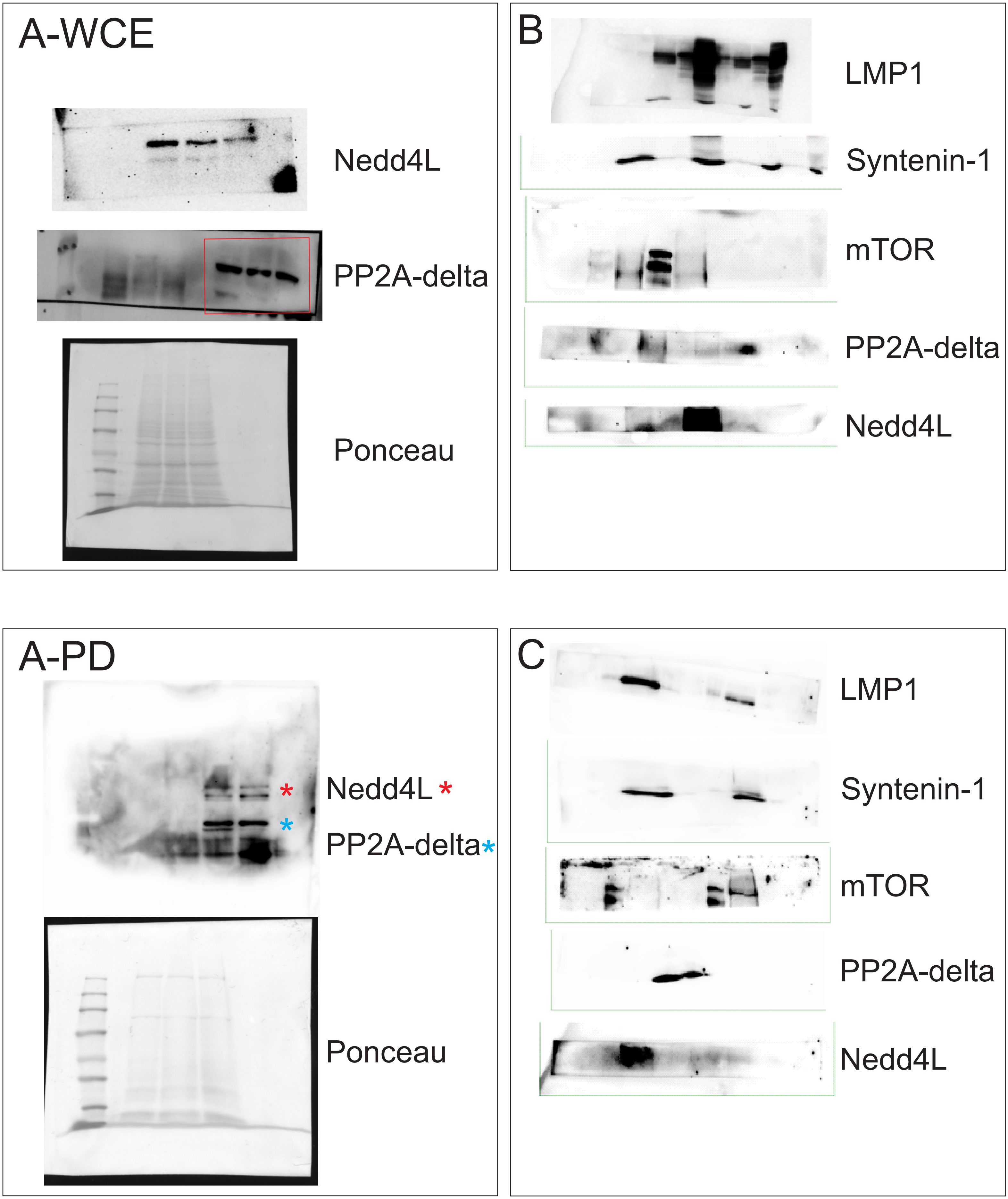

A-WCE
Nedd4L
PP2A-delta
Ponceau
B
LMP1
Syntenin-1
mTOR
PP2A-delta
Nedd4L
A-PD
Nedd4L *
PP2A-delta*
Ponceau
C
LMP1
Syntenin-1
mTOR
PP2A-delta
Nedd4L

Figure 6

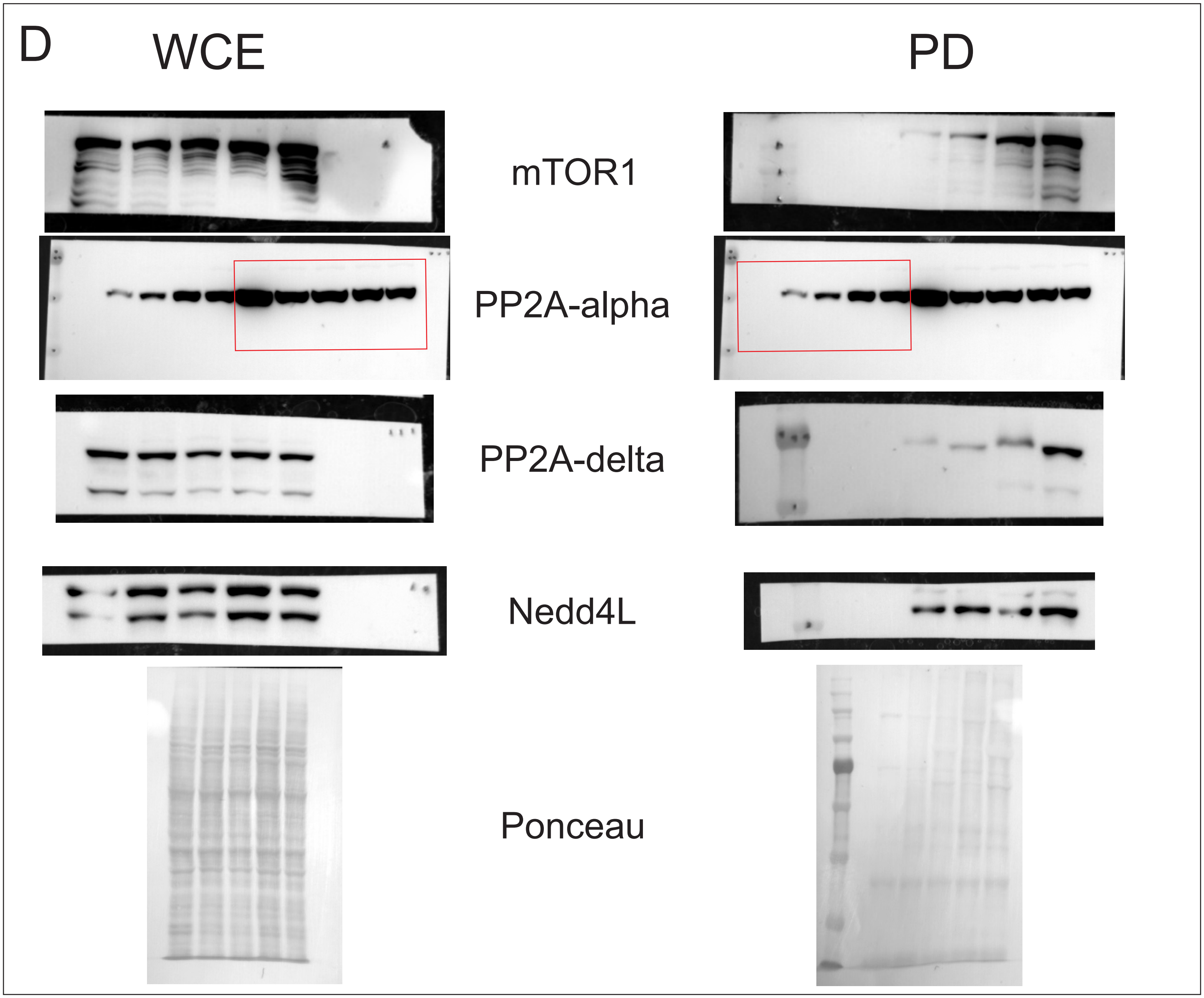
D
WCE
PD
mTOR1
PP2A-alpha
PP2A-delta
Nedd4L
Ponceau

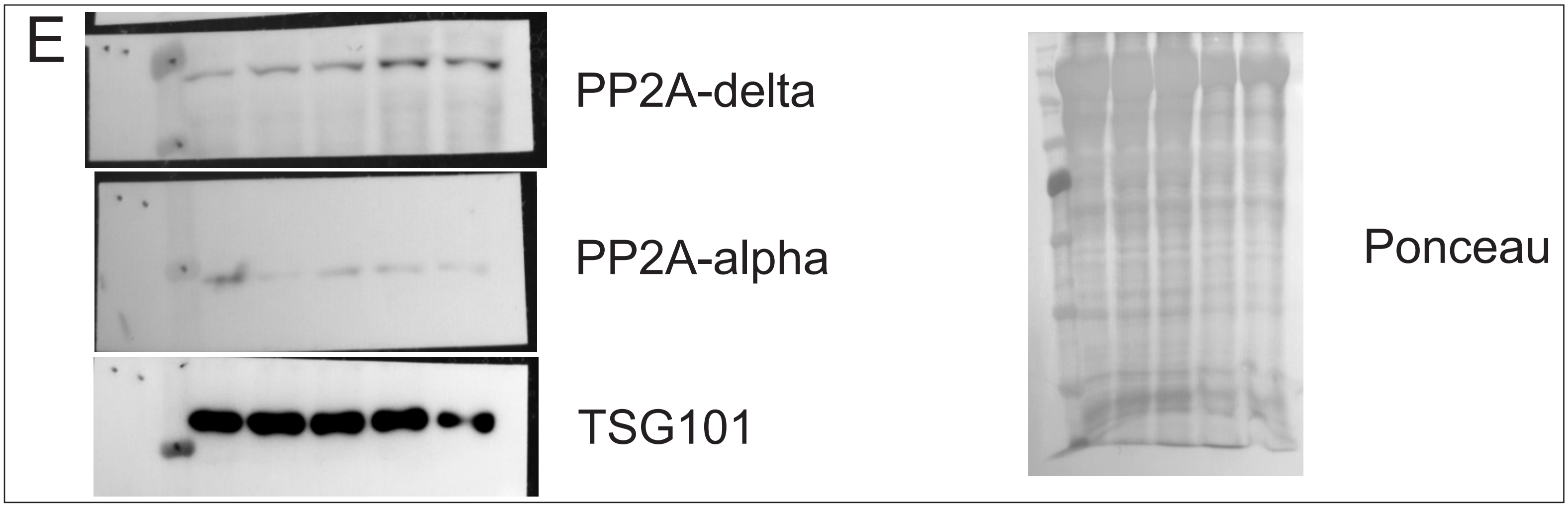
E
PP2A-delta
PP2A-alpha
TSG101
Ponceau
